# Complaint-Directed Mini-Interventions for Depressive Symptoms: A Health Economic Evaluation of Unguided Web-Based Self-Help Interventions Based on a Randomized Controlled Trial

**DOI:** 10.2196/10455

**Published:** 2018-10-01

**Authors:** Ben FM Wijnen, Suzanne Lokman, Stephanie Leone, Silvia MAA Evers, Filip Smit

**Affiliations:** 1 Centre for Economic Evaluation Trimbos Institute Netherlands Institute of Mental Health and Addiction Utrecht Netherlands; 2 Department of Health Services Research Care and Public Health Research Institute School for Public Health and Primary Care Maastricht University Maastricht Netherlands; 3 Department of Public Mental Health Trimbos Institute Netherlands Institute of Mental Health and Addiction Utrecht Netherlands; 4 Department of Clinical Psychology Faculty of Behavioural and Movement Sciences VU University Amsterdam Netherlands; 5 Amsterdam Public Health Research Institute Department of Epidemiology and Biostatistics VU University Medical Center Amsterdam Netherlands

**Keywords:** prevention, depression, internet-based intervention, economic evaluation, quality of life, cost-effectiveness analysis, cost-utility analysis, early medical intervention, cost-benefit analysis

## Abstract

**Background:**

Depression prevention and early intervention have become a top priority in the Netherlands, but with considerable room for improvement. To address this, Web-based complaint-directed mini-interventions (CDMIs) were developed. These brief and low-threshold interventions focus on psychological stress, sleep problems, and worry, because these complaints are highly prevalent, are demonstrably associated with depression, and have substantial economic impact.

**Objective:**

The objective of this economic evaluation was to examine the added value of Web-based, unguided, self-help CDMIs compared with a wait-listed control group with unrestricted access to usual care from both a societal and a health care perspective.

**Methods:**

This health economic evaluation was embedded in a randomized controlled trial. The study entailed 2 arms, in which 3 Web-based CDMIs were compared with a no-intervention waiting-list control group (which received the intervention after 3 months). We conducted measurements at baseline, and at 3 and 6 months. The primary outcome was the rate of responders to treatment on depressive symptoms as measured by the Inventory of Depressive Symptomatology Self-Report (IDS-SR). We estimated change in quality of life by calculating effect sizes (Cohen *d*) for individual pre- and posttreatment IDS-SR scores using a conversion factor to map a change in standardized effect size onto a corresponding change in utility. We calculated incremental cost-effectiveness ratios using bootstraps (5000 times) of seemingly unrelated regression equations and constructed cost-effectiveness acceptability curves for the costs per quality-adjusted life-year (QALY) gained.

**Results:**

Of 329 study participants, we randomly assigned 165 to the CDMI group. At 3 months, the rate of responders to treatment was 13.9% (23/165) in the CDMI group and 7.3% (12/164) in the control group. At 3 months, participants in the CDMI group gained 0.15 QALYs compared with baseline, whereas participants in the control group gained 0.03 QALYs. Average total costs per patient at 3 months were €2094 for the CDMI group and €2230 for the control group (excluding baseline costs). Bootstrapped seemingly unrelated regression equations models resulted in a dominant incremental cost-effectiveness ratio (ie, lower costs and a higher rate of responders to treatment) for the CDMI group compared with the control group at 3 months, with the same result for the costs per QALY gained. Various sensitivity analyses attested to the robustness of the findings of the main analysis.

**Conclusions:**

Brief and low-threshold Web-based, unguided, self-help CDMIs have the potential to be a cost-effective addition to usual care for adults with mild to moderate depressive symptoms. The CDMIs improved health status, while reducing participant health care costs, and hence dominated the care-as-usual control condition. As intervention costs were relatively low, and the internet is readily available in the Western world, we believe CDMIs can be easily implemented on a large scale.

**Trial Registration:**

Netherlands Trial Register NTR4612; http://www.trialregister.nl/trialreg/admin/rctview.asp?TC=4612 (Archived by WebCite at http://www.webcitation.org/6n4PVYddM)

## Introduction

### The Burden of Depression

Globally, more than 300 million people from different age groups have depression [1]. Likewise, depression is the most prevalent psychological disorder in the Netherlands. In 2017, about 550,500 people aged 18 to 65 years had a depression disorder [2]. In addition, slightly more than 1 million people (1,006,700) had subclinical depression (ie, people with the core symptoms of depression otherwise not fulfilling the diagnostic criteria of major depression) [3]. However, depression not only causes individual suffering and loss of quality of life [4], but is also associated with economic costs as a result of health care utilization and reduced productivity owing to absenteeism and lesser efficiency while at work [5]. In 2011, the cost of treatments for depression was €1.6 billion in the Netherlands [6]. This corresponds to 1.8% of the total expenditure on health care. Moreover, a study by Greenberg et al estimated that 48% to 50% of the total costs of depression were related to workplace costs [7].

Given the high prevalence and the chronic character of the condition, prevention and early intervention is important, especially as current psychological and pharmacological interventions have been shown to only moderately reduce the burden of depression [2,8].

### Web-Based Complaint-Directed Mini-Interventions

In the past decades, depression prevention and early intervention has become a top priority in the Netherlands. However, despite evidence that such programs can be effective, there is still considerable room for improvement. Specifically, there is a need for novel interventions that are easily accessible, cheap, and, importantly, suitable for high-risk populations (eg, people with a low socioeconomic status), as the reach of preventive or early mental health interventions is far from optimal among these populations. It is also important that interventions are able to encourage self-management and can be implemented against limited costs. With this in mind, the Web-based complaint-directed mini-interventions (CDMIs) were developed [9]. The unique feature of the CDMIs as an approach for depression is that they were developed by taking into account that symptoms preceding or underlying depression may not be disorder specific (eg, worry) and may also vary by individual, which is in line with recent symptom network and transdiagnostic approaches to mental disorders [10-12]. These brief and low-threshold interventions focus on psychological stress, sleep problems, and worry, because these complaints are highly prevalent, are demonstrably associated with depression, have a substantial economic impact, and are frequently presented to the general practitioner [13-15]. Hence, CDMIs focus on tackling complaints (symptoms) rather than on disorders, which allows each individual to choose the complaint(s) they want to focus on according to their needs. This feature may be advantageous, as it may provide a better fit between what an individual needs and the intervention. In total, 3 different CDMIs were developed: Sleep better, Stress less, and Worry less.

Although the impact of interventions on individual and population health is vital, it is also important to determine the role of different interventions in contributing to other socially desirable goals, such as reducing societal (health care) costs. In addition, due to scarcity of resources for and rising costs of the health care system, economic evidence is becoming increasingly important for decision makers and regulatory bodies. Health economic evaluations aim to provide economic evidence on the costs and effects of (new) health care interventions. In a health economic evaluation, both the costs and effects of 2 (or more) alternative treatments or interventions are compared in a systematic manner. In this way, it is possible to examine which alternative is most efficient and hence provides the best value for money [16]. The use of economic evaluations is required by the National Health Care Institute in the Netherlands [17] and by the National Institute for Health and Care Excellence in the United Kingdom [18]. Treating people in a relatively accessible way in an early stage may prevent them from developing more serious mental disorders (eg, chronic depression), and possibly prevent them from needing high-cost mental health care.

Web-based interventions for depressive symptoms have been shown to be cost effective in the past. For example, McCrone et al assessed the cost effectiveness of computer-delivered cognitive behavioral therapy and concluded that it has a high probability of being cost effective [19]. Moreover, Warmerdam et al evaluated the cost effectiveness of internet-based cognitive behavioral therapy and internet-based problem-solving therapy and concluded that both have a high probability of being cost effective [20]. Both studies, however, examined interventions that were relatively extensive compared with the CDMIs.

### Objective

The aim of this economic evaluation was to examine the added value of the Web-based unguided self-help CDMIs as compared with a wait-listed control group with unrestricted access to usual care from both a societal and a health care perspective. We conducted the health economic evaluation in a sample of adults with mild to moderate depressive symptoms from a societal perspective. In addition, we conducted the analysis from an employers’ perspective to determine the cost and effects associated with CDMIs specifically related to productivity losses in the subsample of people with a paid job.

## Methods

### Parent Randomized Controlled Trial

This economic evaluation was embedded in a randomized controlled trial (see Lokman et al [21]). The study entailed 2 arms in which we compared 3 Web-based CDMIs with a no-intervention waiting-list control group. We used stratified block randomization to ensure that participants were equally distributed over the 3 different CDMIs (ie, Sleep better, Stress less, or Worry less) and 2 levels of education (high: higher vocational or university-level education; or low educated). Measurements were conducted at baseline, and at 3 and 6 months’ follow-up.

### Study Population and Recruitment

Patients were included if they fulfilled the following criteria: (1) at least 18 years of age, (2) access to a computer with an internet connection, (3) sufficient proficiency of the Dutch language, (4) adequate computer skills to participate in the training, and (5) mild to moderate depressive symptoms defined as a score of 14 to 38 on the Inventory of Depressive Symptomatology Self-Report (IDS-SR) [22]. These IDS-SR cutoff scores imply that the CDMIs were used for indicated prevention in subclinical depression and early intervention in mild depression. Participants were excluded if they had suicidal thoughts or plans as measured with item 18 of the IDS-SR. These participants were referred to contact their general practitioner or an anonymous online platform for people with suicidal thoughts. The required sample size for this study to achieve a power of 80% was estimated to be 292 (146 per condition), based on achieving an effect size of 0.33 with a power of .80 and a 2-tailed test with alpha=.05.

Participants were recruited from June 2014 to January 2015 via open recruitment (ie, through relevant websites, messages on social media, and messages in digital newsletters of the Trimbos Institute, Utrecht, the Netherlands). Next, people interested in participation were referred to a special study website where they were given more information about the study and could register to take part in the study by completing a written or an online informed consent form including their name and email address. Applicants were requested to complete the first part of the self-report online baseline questionnaire, which acted as a screening instrument and consisted of the IDS-SR and questions about age, internet access, and computer skills. Eligible participants received the second part of the online baseline questionnaire. To be able to conduct the stratified block randomization, we asked applicants which CDMI they would want to take part in: Sleep better, Stress less, or Worry less. The study was approved by the Medical Ethics Committee of the University Medical Center Utrecht and is registered in the Netherlands Trial Register (NTR): NTR4612. More details can be found in Lokman et al [21].

### Intervention

The 3 CDMIs are unguided, Web-based, self-help interventions to prevent or reduce depressive complaints. As noted in the introduction, symptoms preceding or underlying (a developing) depression may not be disorder specific (eg, worry) and may also vary by individual. Therefore, the CDMIs were developed with a focus on specific complaints rather than being a program targeting a subclinical disorder. This allows each individual to choose the complaint(s) they want to focus on based on their needs. Thus, the CDMIs were developed taking into account that symptoms of depression or a developing depression varies by individual while still serving as an overall approach to combat depressive symptoms.

The CDMIs are therefore complaint focused rather than disorder focused. The content of the CDMIs is largely based on cognitive behavioral techniques but also incorporates elements from solution-focused therapy, mindfulness, and positive psychology. The CDMIs are made up of 3 to 4 modules, with each module consisting of 4 to 6 exercises. Some modules, such as relaxation, are relevant for all 3 complaints (sleep, stress, and worry) and are, therefore, part of all 3 CDMIs. Fixed elements in every CDMI are a home page, a diary, a list of the user’s favorite exercises, an exercise book, a to-do list, and a library. Users were free to choose between the modules and exercises and could work independently through the CDMI, without supervision. Participants received a reminder if they did not log in to the CDMI within 1 week after registration. Participants were advised to spend 2 to 3 hours a week on the CDMI for a period of at least 4 weeks. Multimedia Appendix 1 (adapted from Lokman et al [21]) contains a more detailed description of the CDMIs (including screenshots). All CDMIs were developed by the Trimbos Institute (Netherlands Institute for Mental Health and Addiction).

#### Control Group

Participants randomly assigned to the control group were placed on a waiting list for 3 months with unrestricted care as usual. They were provided access to the CDMI of their choice afterward.

#### Outcomes

The primary outcome of the study was depressive symptoms as measured by the IDS-SR [22]. The IDS-SR consists of 30 items relating to the last 7 days that cover 9 diagnostic symptom domains used to characterize a major depressive episode, as well as items to define melancholic and atypical symptom features, commonly associated symptoms (eg, irritability, anxiety), and features of endogenous symptoms. We chose the IDS-SR as the primary outcome because the study focused on adults with mild to moderate depressive symptoms, and we hypothesized a greater reduction in depressive complaints for the participants using the Web-based CDMIs. Items are scored on a 4-point Likert scale and can be summed to obtain a total score. Scores range from 0 to 84, with higher scores indicating greater depressive symptom severity. For the cost-effectiveness analysis, we used the rate of responders to treatment as the central clinical end term, arbitrarily chosen to ease interpretation of the cost-effectiveness analysis (ie, additional costs per responder instead of additional costs per percentage improvement on IDS-SR scores). Rate of responders to treatment was defined as having a decrease on the IDS-SR scale by 50% (or more) compared with baseline.

Then, we estimated a change in quality of life by calculating effect sizes (Cohen *d*) for individual pre- and posttreatment IDS-SR scores (ie, Cohen *d* = (IDS-SR[TX]–IDS-SR[T0]) / standard deviation of IDS-SR[T0]), where T0 is baseline and TX values are the assessments at the 3- and 6-month follow-ups. Next, we used the conversion factor of Sanderson and colleagues to map a change in standardized effect size onto a corresponding change in utility for people with depression [23]. This conversion factor entails the average difference in utility that is associated with a difference of 1 effect size. Utilities represent the value of a particular health state on a scale anchored at 0 and 1, in which 0 means death and 1 means perfect health. We then used the utilities to calculate quality-adjusted life-years (QALYs) by multiplying them by the time spent in that particular health state [16].

#### Costs

We used the Dutch guidelines for economic evaluations [24] and the Consolidated Health Economic Evaluation Reporting Standards [25] to conduct the economic evaluation and to report the outcomes. Conforming to the Dutch guidelines, we took a societal perspective, in which all relevant costs for society should be taken into consideration. Based on this guidance, these costs entail patient and family costs (eg, travel costs, home care, informal care) and productivity losses (ie, presenteeism and absenteeism from work).

We measured (health care) resource use, costs for patient and family, and productivity losses using the Trimbos/iMTA Questionnaire for Costs Associated With Psychiatric Illness [26].

We distinguished 4 cost categories: intervention costs, health care sector costs, costs for patient and family, and costs owing to productivity losses. Intervention costs were based on the total number of accounts per year, the costs for hosting and updating the website, and costs for support by a helpdesk that could be reached by phone. To value cost items, we used standardized cost prices from the Dutch manual for costing [24]. If those were not available, we used mean cost prices from providers. To determine the costs of drugs, we asked participants for how many days they had used drugs for depression, problems sleeping, or anxiety. Next, we assigned monetary values based on an average cost price per day for depression, sleep, or anxiety disorders separately as determined by average cost per day (using data from the Dutch Healthcare Institute [27]) for a selection of the most prescribed drugs for each category in combination with their recommended daily dose (using data from the Dutch Healthcare Institute [28]).

As recommended in the Dutch guidelines, productivity losses were estimated using the friction cost approach. The friction cost approach entails the calculation of productivity losses only during a prespecified friction period (85 days according to the Dutch guidelines) [24]. This period is supposed to be the time until another worker from the pool of unemployed has fully replaced the individual who is absent due to an illness. We considered both absenteeism (absence from work due to sickness) and presenteeism (reduced productivity). Furthermore, we valued patients’ time and informal care using the proxy good method, using the average hourly wage of domestic help as a proxy.

All costs were indexed for the year 2016. Given the follow-up of the study, no discounting was performed.

#### Analyses

We carried out all analyses while adhering to the intent-to-treat principle; that is, we analyzed all participants as randomized provided that their baseline data were complete. For these analyses, we imputed missing values at the follow-up measurements using multiple imputation (5 times). Imputation for total costs and IDS-SR scores was based on age, sex, group, baseline IDS-SR score, baseline Jenkins Sleep Evaluation Questionnaire (JSEQ) score, baseline Perceived Stress Scale (PSS) score, baseline Generalized Anxiety Disorder 7-item (GAD-7) scale score, and baseline health care, patient and family, and productivity costs (for cost data only). To account for nonnormality of the data, we used predictive mean matching, in which real observed values from similar cases are imputed instead of imputing regression estimates [29,30].

We used rate of responders to treatment, as determined by at least a 50% decrease in IDS-SR scores, to calculate an incremental cost-effectiveness ratio (ICER) by dividing the difference in costs by the difference in rate of responders to treatment between both groups. This resulted in the additional costs per extra responder. In addition, we used QALY estimates to calculate the incremental cost-utility ratio (ICUR) by dividing the difference in costs by the difference in QALY between both groups. This way, the ICUR represents the additional costs per QALY gained. To investigate the uncertainty around the ICER and ICUR, we used nonparametric bootstrapping (5000 times). Bootstrapping is a nonparametric way to repeatedly simulate an analysis by resampling, with replacement, from the observed data [31]. We bootstrapped (5000 times) seemingly unrelated regression equations (SUREs) to allow for correlated residuals of the cost and utility equations and to account for baseline differences in productivity costs. Next, we constructed cost-effectiveness acceptability curves for the costs per QALY gained, in which the likelihood that the CDMIs are cost effective is presented given several willingness-to-pay ceilings. A report from the Dutch Council for Public Health and Health Care provided guidance on the ceiling ratios for a QALY for diseases defined by their disability weight. Based on this report, the ceiling ratio can be roughly estimated to be €20,000 to €80,000 per QALY depending on the severity of the disease or disorder [32], and since we were looking at subclinical and mild manifestations of depression, this would put the willingness-to-pay ceiling at €20,000 per QALY gained.

We present comparative results for 3 months’ follow-up, as the control group received the CDMIs after 3 months of follow-up, which hampers interpretation at 6 months’ follow-up. We used results at 6 months only to check whether effects at 3 months were sustained. All analyses were carried out using Stata 14 (StataCorp LLC).

#### Sensitivity Analyses

We conducted sensitivity analyses to assess the impact of certain assumptions on the results presented in the base case for 3 months’ follow-up (as this was the end of the comparative phase). First, we performed a subgroup analysis of only participants with a paid job to determine the effect of CDMIs on productivity losses (due to both absenteeism and presenteeism) in relation to the intervention costs (ie, investment costs from an employers’ perspective). Second, we conducted an analysis without baseline adjustments (ie, as the base case analyses were based on baseline-adjusted estimates) to determine the impact of these adjustments on the ICERs and ICURS. Third, we performed analyses from a health care perspective, which excluded all patient and family costs and productivity losses. We did this because the health care perspective is a dominant perspective in health economics and is recommended as the main perspective in certain countries, for example, in the United Kingdom by The National Institute for Health and Care Excellence [33]). Fourth, although opportunity costs for participants (their time spent using the CDMIs) should not be included according to the Dutch guidelines, we conducted an additional sensitivity analysis in which we incorporated these costs in the intervention costs of these self-help CDMIs to see whether this would lead to different conclusions. Fifth, given the relatively large dropout at T1 (3 months), we conducted additional sensitivity analyses in which we used 2 different imputation techniques. First, we used regression-based imputation, in which we checked which predictors were significantly associated with dropout at T1 and which predictors were significantly associated with the primary outcome. We then used these variables in a linear regression to impute missing values [34]. Second, we used a simple imputation based on last observation carried forward per patient. Using different imputation techniques, we were able to determine whether the use of a different approach would have affected our base case results.

## Results

### Sample at Baseline

In total, we included 329 participants in the study, of whom we randomly assigned 165 to the CDMI condition and 164 to the (waiting-list) control condition. Participants in the CDMI group were distributed among the 3 interventions as follows: Sleep better, n=59 (35.7%); Stress less, n=45 (27.3%); and Worry less, n=61 (37.0%). During the 3-month intervention period, the participants logged in a median of 3 times (range 0-166, interquartile range 5). After 3 months, participants in the waiting list were allowed access to the interventions and were distributed in a similar pattern: Sleep better, n=60 (36.7%); Stress less, n=43 (26.2%); and Worry less, n=61 (37.2%). Table 1 presents the baseline characteristics of all participants.

### Loss to Follow-Up

At 3 months, 68 participants were lost to follow-up in the CDMI group (41.2%) and 24 participants in the control group (14.6%), which was statistically significant (χ^2^_1_=28.8, *P*<.001). Hence, we performed a sensitivity analyses using covariates significantly associated with dropout at 3 months. At 6 months, 97 participants were lost to follow-up in the CDMI group (58.8%) and 82 in the control group (50.0%) cumulatively. This resulted in 68 participants with complete follow-up in the CDMI group and 82 in the control group.

### Clinical Outcomes

At 3 months’ follow-up, the rate of responders to treatment was 13.9% (23/165) in the CDMI group and 7.3% (12/164) in the control group. At 6 months’ follow-up rates of responders to treatment were 18.8% (31/165) and 11.6% (19/164), respectively. When looking at quality of life, participants in the CDMI group gained 0.15 QALY at both 3 and 6 months’ follow-up compared with baseline, whereas participants in the control group gained 0.03 QALY at 3 months’ follow-up and 0.16 QALY at 6 months’ follow-up (see Table 2 [23]). A more detailed analysis on the clinical outcomes can be found in Lokman et al [21]. In short, Lokman et al demonstrated a significant reduction in depressive symptoms for participants in the intervention group compared with participants in the waiting-list control group after 3 months’ follow-up. Furthermore, significant effects were observed for sleep problems, worry, anxiety, and well-being [21].

### Costs

At baseline, productivity losses were higher in the CDMI group (see Table 3). Hence, we adjusted bootstrapped SURE models to correct for this baseline difference.

Average total costs per patient (intervention costs, health care costs, patient and family costs, and productivity losses) during the 3-month follow-up were €2094 for the CDMI group and €2230 for the control group (excluding baseline costs; Table 4). At 6 months’ follow-up, total costs were €3643 for the CDMI group and €3534 for the control group. No large cost differences were demonstrated between the 2 groups. We estimated intervention costs based on the yearly number of accounts (5000) at €3.90 per participant. When including the participants’ time costs (opportunity costs), intervention costs were €52.9 per participant.

### Incremental Cost-Effectiveness Ratios

**Figure 1:**
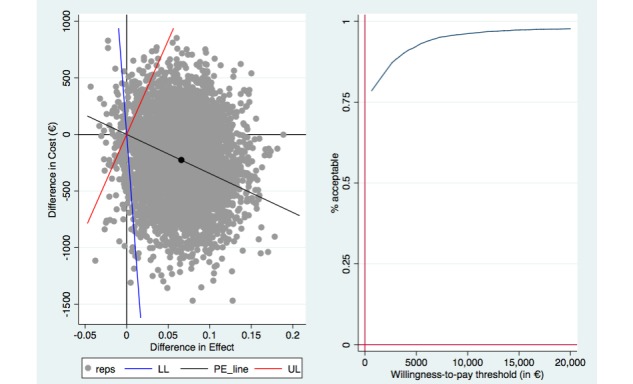
Cost-effectiveness plane (left) and cost-effectiveness acceptability curve (right) of rates of responders to treatment at 3-month follow-up (costs per extra responder). LL: lower limit of the 95% CI; PE: mean incremental cost-effectiveness ratio (ICER); reps: ICER replication; UL: upper limit of the 95% CI.

**Figure 2:**
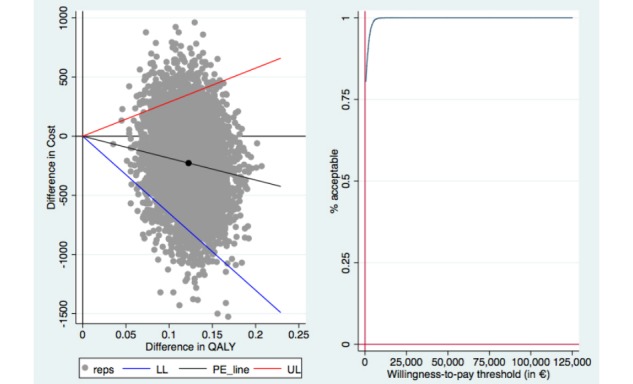
Cost-effectiveness plane (left) and cost-effectiveness acceptability curve (right) of quality-adjusted life-year (QALY) gain (costs per QALY gained) after 3 months. LL: lower limit of the 95% CI; PE: mean incremental cost-utility ratio (ICUR); reps: ICUR replication; UL: upper limit of the 95% CI.

Bootstrapped SURE models, in which we made a baseline adjustment regarding productivity losses, resulted in a dominant ICER (ie, lower costs and a higher rate of responders to treatment) for the CDMI group compared with the control group at 3 months’ follow-up (see Figure 1). Likewise, the ICURs in terms of QALY gain were dominant (ie, lower costs and increases in utility) when compared with the control group at 3 months’ follow-up (see Figure 2).

**Table 1:** Demographic characteristics and clinical information on participants in the complaint-directed mini-intervention (CDMI) and control groups at baseline (N=329).

Characteristic		CDMI group (n=165)	Control group (n=164)
Age (years), mean (SD), range		42.85 (12.83), 18-76	43.65 (13.05), 18-81
**Sex, n (%)**			
	Female	122 (73.9)	127 (77.4)
	Male	43 (26.1)	37 (22.6)
**Marital status, n (%)**			
	Single	83 (50.3)	84 (51.2)
	Living with partner	82 (49.7)	80 (48.8)
**Nationality, n (%)**			
	Dutch	2 (1.8)	4 (2.4)
	Other	160 (97.6)	163 (98.2)
**Living arrangement, n (%)**			
	Alone	40 (24.2)	39 (23.8)
	With other	125 (75.8)	125 (76.2)
**Education level, n (%)**			
	Low	50 (30.3)	48 (29.3)
	High	115 (69.7)	116 (70.7)
**Employment, n (%)**			
	Paid	116 (70.3)	117 (71.3)
	Unpaid	49 (29.7)	47 (28.7)
**Duration of complaints (years), n (%)**			
	<1	59 (35.8)	63 (38.4)
	≥1	106 (64.2)	101 (61.6)
**Severity of complaints, n (%)**			
	Low	68 (41.2)	83 (50.6)
	High	97 (58.8)	81 (49.4)
**Evaluation scores, mean (SD)**			
	Sleep (Jenkins Sleep Evaluation Questionnaire)	11.61 (5.42)	11.21 (5.34)
	Stress (Perceived Stress Scale)	21.82 (5.86)	21.48 (5.37)
	Worry (Penn State Worry Questionnaire)	37.76 (9.26)	38.28 (9.61)
	Anxiety (Generalized Anxiety Disorder 7-item)	10.09 (4.16)	10.04 (3.73)

**Table 2:** Rates of responders to treatment and utilities at baseline, and 3- and 6-month follow-ups by group (N=329) for the complaint-directed mini-intervention (CDMI) and control groups.

Measure	CDMI group (n=165)				Control group (n=164)		
	Baseline	3 months	6 months	Baseline		3 months	6 months
Rates of responders to treatment based on IDS-SR^a^, n (%)	N/A^b^	23 (13.9)	31 (18.8)	N/A		12 (7.3)	19 (11.6)
Utilities and quality-adjusted life-years^c^ gained, mean (SD)	N/A	0.15 (0.02)	0.15 (0.02)	N/A		0.03 (0.02)	0.16 (0.01)

^a^IDS-SR: Inventory of Depressive Symptomatology Self-Report.

^b^N/A: not applicable.

^c^Calculated using a translation factor to transform differences in effect size to changes in utility for people with depression developed by Sanderson et al [23].

**Table 3:** Average per-patient baseline costs for the complaint-directed mini-intervention (CDMI) and control groups^a^.

Cost type	CDMI group (n=165)		Control group (n=164)	
	Mean cost (€)	95% CI	Mean cost (€)	95% CI
Total health care costs	197	162-232	243	146-340
Total patient and family costs	87	16-159	53	15-91
Total productivity losses	729	520-937	582	387-778

^a^1 month; indexed for the year 2016.

**Table 4:** Overview of total costs during the 3-month follow-up for the complaint-directed mini-intervention (CDMI) and control groups^a^.

Cost type		CDMI group (n=97)		Control group (n=140)	
		Mean cost (€)	95% CI	Mean cost (€)	95% CI
Intervention costs		4	—	0	—
**Health care costs**					
	General practitioner visits	111	—	89	—
	General practitioner support	8	—	5	—
	Social worker	10	—	21	—
	Psychologist	84	—	84	—
	Physiotherapist	52	—	55	—
	Psychiatry	91	—	75	—
	Other visits^b^	40	—	68	—
	Medication	151	—	224	—
	Total health care costs^c^	519	428-611	600	458-741
**Patient and family costs**					
	Home care	3	—	39	—
	Special home care^b^	19	—	1	—
	Informal care	100	—	83	—
	Total patient and family costs^c^	114	77-151	126	38-214
**Productivity losses**					
	Absenteeism paid work	943	—	911	—
	Presenteeism paid work	560	—	625	—
	Total production losses^c^	1461	1087-1835	1504	984-2024
Total costs after 3 months^c^		2094	1692-2496	2230	1679-2979

^a^Indexed for the year 2016.

^b^Includes alternative healing and self-support groups.

^c^Totals and subtotals based on multiple imputation estimates (CDMI group: n=165; control group: n=164).

### Sensitivity Analyses

Limiting the analysis to patients with a paid job resulted in a dominant ICER at 3 months’ follow-up (ie, lower costs and a higher rate of treatment response). When excluding baseline corrections, results were similar to the base case analyses (ie, dominant ICER at 3 months’ follow-up). Including opportunity costs for patients also did not affect the ICER at 3 months. We found a dominant ICER when taking the health care perspective at 3 months’ follow-up.

Looking at the costs per QALY gained, excluding patients without a paid job or an analysis without baseline correction resulted in a dominant ICUR for CDMI compared with the control group at 3 months’ follow-up (ie, lower costs and increase in QALYs). Including opportunity costs for patients resulted in a dominant ICUR. From a health care perspective, correspondingly, we found a dominant ICUR at 3 months’ follow-up.

**Table 5:** Cost-effectiveness analyses and sensitivity analyses for rate of responders to treatment and quality-adjusted life-years (QALYs) at 3-month follow-up.

Analysis		Incremental cost (€)	Incremental effect	Mean ICER^a^	Distribution of 5000 bootstrap simulated ICERs			
					NE^b^	SE^c^ (dominant)	SW^d^	NW^e^ (inferior)
**Cost effectiveness, rate of responders to treatment**								
	Main analysis	–225	0.07	Dominant	25.2	72.4	1.6	0.8
	Only including participants with paid job	–309	0.12	Dominant	25.0	74.6	0.3	0.1
	Analysis without baseline adjustments	–131	0.07	Dominant	33.7	63.9	1.4	1.0
	Health care perspective	–78	0.07	Dominant	17.1	80.5	1.9	0.5
	Including opportunity costs for participants	–225	0.07	Dominant	25.2	72.4	1.6	0.8
	Regression-based imputation	–127	0.07	Dominant	34.4	63.3	1.3	1.0
	Last observation carried forward	–784	0.05	Dominant	4.5	90.5	4.7	0.3
**Cost utility, QALYs**								
	Main analysis	–225	0.12	Dominant	24.7	75.3	0	0
	Only including participants with paid job	–312	0.15	Dominant	24.9	75.1	0	0
	Analysis without baseline adjustments	–131	0.12	Dominant	34.7	65.3	0	0
	Health care perspective	–85	0.12	Dominant	16.1	83.9	0	0
	Including opportunity costs for participants	–228	0.12	Dominant	25.7	74.3	0	0
	Regression-based imputation	–130	0.12	Dominant	35.1	64.9	0	0
	Last observation carried forward	–785	0.06	Dominant	4.8	95.0	0.2	0.1

^a^ICER: incremental cost-effectiveness ratio.

^b^NE: northeast quadrant (the intervention was more effective and more costly than usual care).

^c^SE: southeast quadrant (the intervention was more effective and less costly than usual care).

^d^SW: southwest quadrant (the intervention was less effective and less costly than usual care).

^e^NW: northwest quadrant (the intervention was less effective and more costly than usual care).

Using covariates significantly associated with dropout at 3 months (ie, baseline GAD-7 score and age) and covariates significantly associated with the primary outcome of rate of responders to treatment on the IDS-R (ie, condition, paid work, baseline JSEQ score, and baseline PSS score) to impute missing values resulted in a dominant ICER and ICUR for CDMI compared with the control group at 3 months’ follow-up. Using last observation carried forward to impute missing values also resulted in a dominant ICER and ICUR for CDMI compared with the control group at 3 months’ follow-up. All in all, the sensitivity analyses attested to the robustness of the findings of the main analysis (see Table 5).

## Discussion

### Principal Findings

This study examined the cost effectiveness of Web-based CDMIs in adult patients with mild to moderate depressive symptoms in comparison with a wait-listed control group with unrestricted access to usual care in the Netherlands. The study had a follow-up at 3 months and in the experimental arm of the trial an extended follow-up at 6 months to see whether effects were sustained over time. The CDMI consisted of 3 different interventions (Sleep better, Stress less, and Worry less). Patients in the waiting-list control group were given access to the CDMIs after 3 months’ follow-up. When looking at the rate of responders to treatment (defined as a 50% reduction in IDS-SR depressive symptoms), we found a dominant ICER at 3 months, implying that the CDMIs provided lower costs for better rates of responders to treatment. Looking at costs per QALY gained, we found a dominant ICER at 3 months, implying lower costs and increased QALYs. For both outcomes, results were sustainable over 6 months, particularly given the steady increase in the rate of responders to treatment in the CDMI group at 6 months and the increase in this responder rate in the control group at 6 months (after giving them access to the intervention at 3 months. Sensitivity analyses showed that results were robust to different assumptions, perspectives, or the way missing data was handled. Hence, this study demonstrated that it is possible to use an easily accessible and economically affordable intervention to improve participants’ health status in a cost-effective manner.

### Evidence in Context

A recent systematic review looking specifically at internet- and mobile-based interventions targeting depression highlighted the potential of those interventions to be cost effective, with cost-effectiveness ratios similar to those reported for face-to-face psychotherapy and antidepressant drug treatment [35]. In this review, of the 14 e-interventions, 6 were deemed cost effective, 5 were not cost effective, and 2 were undecided.

A systematic review looking at economic evaluations of internet interventions for mental health concluded that guided internet interventions for, among others, depression and anxiety demonstrated higher probabilities of being cost effective [36]. However, the evidence for unguided internet interventions for depression was less convincing.

A study examining the cost effectiveness of a Web-based self-help intervention aimed at enhancing well-being by fostering positive emotions and stimulating positive functioning demonstrated reduced depressive symptoms, although at higher costs, leading to unfavorable cost-effectiveness ratios [37]. The authors emphasized the importance of adherence to maintain long-term effects and possibly increase the cost effectiveness.

In terms of feasibility, Griffiths and Christensen [38] evaluated 2 community-based internet programs in the treatment of depression and concluded that these intervention programs could be delivered effectively over the internet. Furthermore, they emphasize the importance of using the internet as a more accessible alternative than face-to-face mental health services, especially in rural areas [38]. CDMIs are well suited to prevent health status deterioration at an early stage, especially in the current climate of increasing emphasis on self-reliance and self-management.

### Strengths and Limitations

This study was not without limitations. First, the follow-up of the trial was 3 months after baseline, which did not provide hard evidence for the longer-term effects. However, in the extended follow-up of the experimental arm, we could see that the effects (rate of responders to treatment and QALY gains) were maintained at 6 months. In addition, at the 6-month follow-up, the costs were relatively lower than those at 3 months (for both groups), suggesting that health care resource use may have decreased. Second, dropout rates were relatively large, which made imputation of missing values necessary. Although high dropout rates are a problem often encountered in electronic health (eHealth) trials [39], one should always carefully consider imputation techniques, especially in the context of substantial dropout. However, different imputation techniques led to comparable results, attesting to the robustness of the main analysis. Third, given the nature of the intervention, participants could not be blinded. This may have biased participants in one way or another (eg, placebo effect). Fourth, because we recruited participants mainly by means of internet-based recruitment avenues, it is possible that we missed some potential participants. For example, we may have missed participants who are less likely to engage in social media. Fifth, although participants were free to find additional care themselves, we did not provide the control condition with any intervention. This may have caused an overestimation of the effects of the CDMIs. Sixth, given the focus of each CDMI, some of the content differed between the 3 CDMIs, although there was also overlap in content. The CDMIs can be seen as an overall intervention approach that aims to target depressive complaints, but one in which participants are able to choose the CDMI they want to use based on their personal needs, and they do not have to use CDMIs that are not relevant to their situation. As a result, each CDMI may target depressive complaints differently. This would be an interesting avenue to explore in future to gain insight into the mechanisms of change. Seventh, the generalizability of the findings to men and those with other educational levels remains to be determined, as mainly highly educated female participants were included in the trial. However, this selected group of participants may well reflect the composition of the target group that will be reached after implementation of the CDMIs.

### Conclusion

This study demonstrated that the brief and low-threshold Web-based, unguided, self-help CDMIs have the potential to be a cost-effective addition to usual care for adults with mild to moderate depressive symptoms. The CDMIs were shown to improve health status, while at the same time reducing health care costs of participants, and hence dominating the care-as-usual control condition. As intervention costs were relatively low, and the internet is nowadays readily available in the Western world, we believe the CDMIs can be easily implemented on a large scale. Future research should aim at increasing the reach of the intervention and determining whether the intervention is indeed more likely to reach people with a low socioeconomic status. Related to this matter, integration of the CDMIs into primary care may be a useful next step, as this would allow the CDMIs to be offered with some guidance from the general practice nurse, possibly boosting effectiveness and adherence. Regarding adherence, future research may also focus on the impact of reminder systems incorporated into the CDMIs.

## Appendix

Multimedia Appendix 1 Detailed insights of complaint-directed mini-intervention (CMDI) including screenshots.

Multimedia Appendix 2 CONSORT‐EHEALTH checklist (V 1.6.1).

## Abbreviations

CDMI: complaint-directed mini-intervention
eHealth: electronic health
GAD-7: Generalized Anxiety Disorder 7-item
ICER: incremental cost-effectiveness ratio
ICUR: incremental cost-utility ratio
IDS-SR: Inventory of Depressive Symptomatology Self-Report
JSEQ: Jenkins Sleep Evaluation Questionnaire
PSS: Perceived Stress Scale
QALY: quality-adjusted life-years
SURE: seemingly unrelated regression equations
