# Supplementary material for: Complaint-Directed Mini-Interventions for Depressive Symptoms: A Health Economic Evaluation of Unguided Web-Based Self-Help Interventions Based on a Randomized Controlled Trial
Source: J Med Internet Res. 2018 Oct 1;20(10):e10455. doi: 10.2196/10455 (PMC6231789; doi:10.2196/10455)
Supplement: Multimedia Appendix 1 [file jmir_v20i10e10455_app1.pdf]

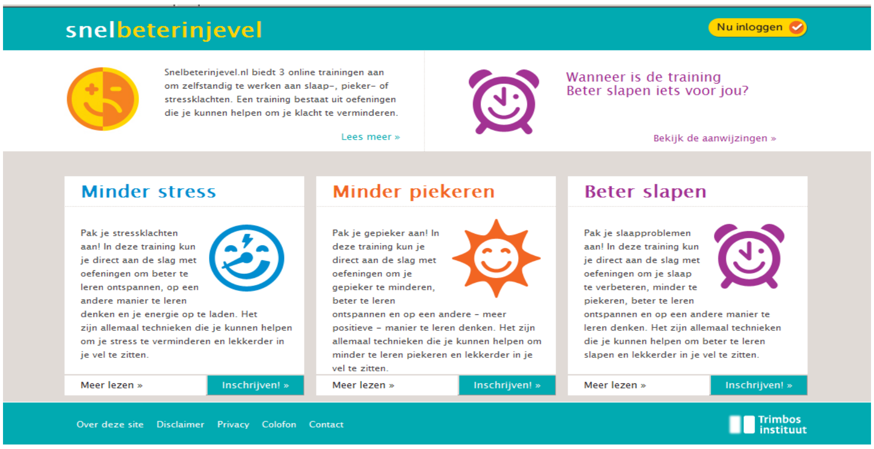

Screenshot of the CDMI homepage

CDMI ‘Sleep Better’
This CDMI is for people who experience sleep problems, like having difficulty falling asleep, frequently waking up during the night or waking up too early. Participants learn to manage factors that influence their sleep negatively and learn skills to positively influence their sleeping behaviour.

The training consists of 4 modules/ topics which include a total of 19 exercises:

- Sleeping habits: 5 exercises that aim to provide more insight into your sleeping habits and learn skills to break bad sleeping
- habits (e.g. check your bedroom).
- Relaxation: 5 exercises to learn relaxation techniques (e.g. progressive relaxation).
- Worry less: 5 exercises aimed at acquiring skills to reduce worry or ruminative thoughts (e.g. 15 minute ‘worry time’).
- Learn to think differently (about sleep): 4 exercises to learn to recognise and change dysfunctional attitudes about sleep.

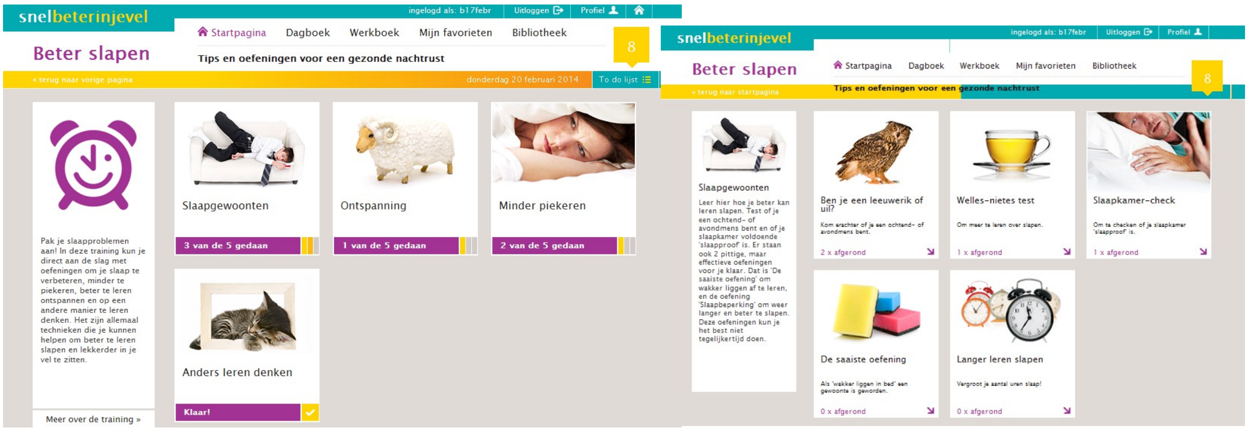

Screenshots of the CDMI Sleep Better

CDMI ‘Worry Less’
This CDMI is for people who tend to worry a lot and want to change this behaviour. The training consists of 4 modules/ topics that include a total of 18 exercises:

- Positive thinking: 4 exercises focused on learning a positive way of thinking (e.g. ‘give yourself a compliment’).

- Worry less: 5 exercises aimed at acquiring skills to reduce worry or ruminative thoughts (e.g. 15 minute ‘worry time’).
- Relaxation: 5 exercises to learn relaxation techniques (e.g. progressive relaxation).
- Learn to think differently: 4 exercises to learn to recognise and change dysfunctional attitudes.

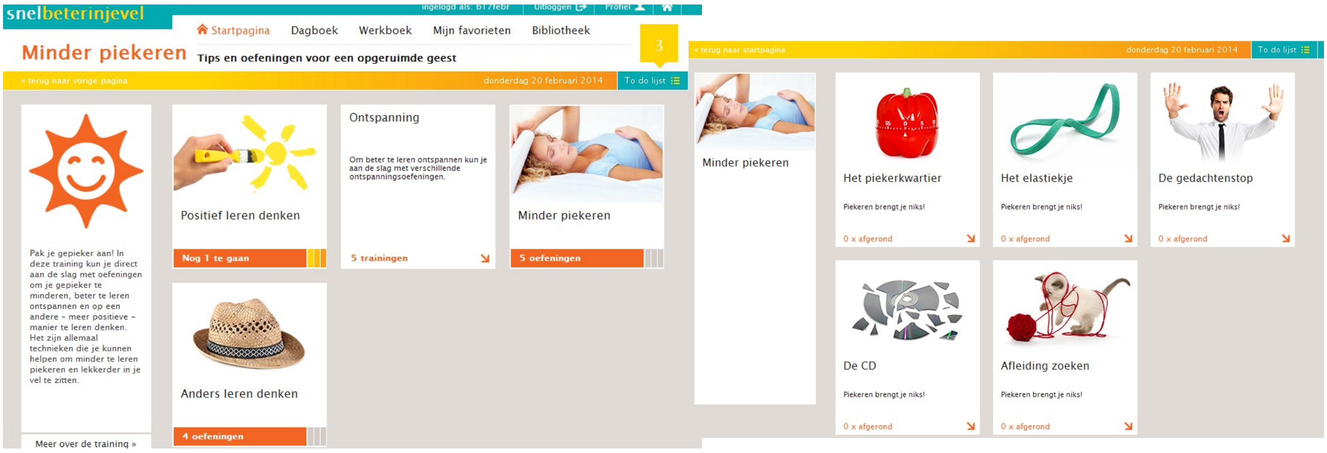

Screenshots of the CDMI Worry Less

CDMI ‘Stress Less’

This CDMI is for people who experience (high levels of) stress. The training consists of 3 modules that include a total of 15 exercises:

- Learn to think differently: 4 exercises to learn to recognise and change dysfunctional attitudes.
- Relaxation: 5 exercises to learn relaxation techniques (e.g. progressive relaxation).
- Boost your energy: 6 exercises aimed at recharging yourself and handling stress (e.g. note 3 positive things).

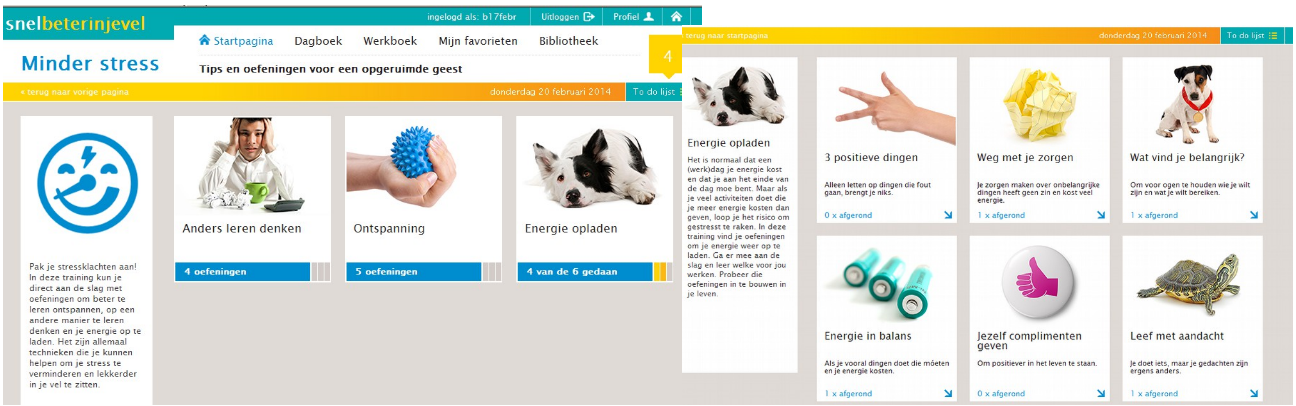

Screenshots of the CDMI Stress Less
